# Enhancing the Thermo-Stability and Anti-Bacterium Activity of Lysozyme by Immobilization on Chitosan Nanoparticles

**DOI:** 10.3390/ijms21051635

**Published:** 2020-02-27

**Authors:** Yanan Wang, Shangyong Li, Mengfei Jin, Qi Han, Songshen Liu, Xuehong Chen, Yantao Han

**Affiliations:** Department of Pharmacology, School of Basic Medicine, Qingdao University, Qingdao 266071, China; sunshine4581@163.com (Y.W.); jinmengfei678@163.com (M.J.); xiaoyu19990727@163.com (Q.H.); liusongshen@126.com (S.L.);

**Keywords:** immobilization, chitosan nanoparticles, lysozyme, anti-bacterium activity

## Abstract

The recent emergence of antibiotic-resistant bacteria requires the development of new antibiotics or new agents capable of enhancing antibiotic activity. Lysozyme degrades bacterial cell wall without involving antibiotic resistance and has become a new antibacterial strategy. However, direct use of native, active proteins in clinical settings is not practical as it is fragile under various conditions. In this study, lysozyme was integrated into chitosan nanoparticles (CS-NPs) by the ionic gelation technique to obtain lysozyme immobilized chitosan nanoparticles (Lys-CS-NPs) and then characterized by dynamic light scattering (DLS) and transmission electron microscopy (TEM), which showed a small particle size (243.1 ± 2.1 nm) and positive zeta potential (22.8 ± 0.2 mV). The immobilization significantly enhanced the thermal stability and reusability of lysozyme. In addition, compared with free lysozyme, Lys-CS-NPs exhibited superb antibacterial properties according to the results of killing kinetics in vitro and measurement of the minimum inhibitory concentration (MIC) of CS-NPs and Lys-CS-NPs against *Pseudomonas aeruginosa* (*P. aeruginosa*), *Klebsiella pneumoniae* (*K. pneumoniae*), *Escherichia coli* (*E. coli*), and *Staphylococcus aureus* (*S. aureus*). These results suggest that the integration of lysozyme into CS-NPs will create opportunities for the further potential applications of lysozyme as an anti-bacterium agent.

## 1. Introduction

Lysozyme degrades certain microorganisms by hydrolyzing the β-1,4 glycosidic linkages between N-acetylmuramic acid and N-acetylglucosamine; these monosaccharides are part of the peptidoglycan of cell wall structure of Gram-positive bacteria [1,2,3]. A wide range of applications for lysozyme has been developed, such as the preservation of food products, potential antimicrobial agent, clinical diagnosis of diseases, antineoplastic agents, etc. [4,5,6,7]. Additionally, the anti-inflammatory, antibacterial, and antineoplastic activities make lysozyme a promising candidate in the application of pharmacological functions, and thus it has become an interesting research area [8].

It is well known that the overuse and misuse of antibiotics causes bacterial resistance to antibiotics, including resistant *P. aeruginosa*, *S. aureus*, and *K. pneumoniae*. Infection with these resistant bacteria can lead to serious life-threatening diseases, such as endocarditis, pneumonia, and sepsis, whereas the lack of effective antibiotics leads to a higher mortality each year [9,10,11]. New antibiotics and other agents that can enhance the antibiotic activity have been developed to address the problem of drug-resistant bacterial infections [12,13]. However, most of these new strategies are unsafe and ineffective in defense against drug-resistant bacterial infections. Thus, it is crucial to exploit new strategies including either effective antibiotic agents or new methods capable of enhancing antibiotic activity. Lysozyme, exerting antibacterial activity mainly through a mechanism involving membrane disruption, is unlikely to develop bacterial resistance and has been exploited as a novel antibacterial strategy [14]. By contrast, the practical application of free lysozyme is quite limited by some problems related to instability against heating and proteolytic load, and its low reusability. In addition, the efficacy of lysozyme is limited to Gram-positive bacteria due to the property of lysozyme targeting the β-1,4 glycosidic linkages between *N*-acetylglucosamine and *N*-acetylmuraminic acid of peptidoglycan. It has been reported that modifying lysozyme can change this limitation, including chemical, physical, and biological changes [15,16]. Among all the strategies, the immobilization of enzymes has attracted considerable interest [17]. Chitosan, a natural biopolymer, has become a suitable substrate for enzymatic immobilization due to its a unique set of properties such as low cost, nontoxicity, biodegradability into harmless products, great biocompatibility, and the ability to load large amounts of enzymes [18,19]. Furthermore, chitosan performs antimicrobial action in various microorganisms without increasing resistance [20].

The aim of this work was to immobilize free lysozyme on chitosan nanoparticles (CS-NPs) to enhance its characteristics. The immobilized lysozyme significantly improved its properties and exhibited greater resistance to high temperature and hold reusability comparing with free enzyme. Moreover, we compared the antibacterial activities of free lysozyme and immobilized lysozyme, showing that immobilized lysozyme exhibited more effective antibacterial activity against *P. aeruginosa*, *K. pneumoniae*, *E. coli*, and *S. aureus*. The disruption of biofilm greatly increased the sensitivity of various bacteria to antibiotics.

## 2. Results and Discussion

### 2.1. Synthesis and Morphology of Chitosan Nanoparticles

**Figure 1:**
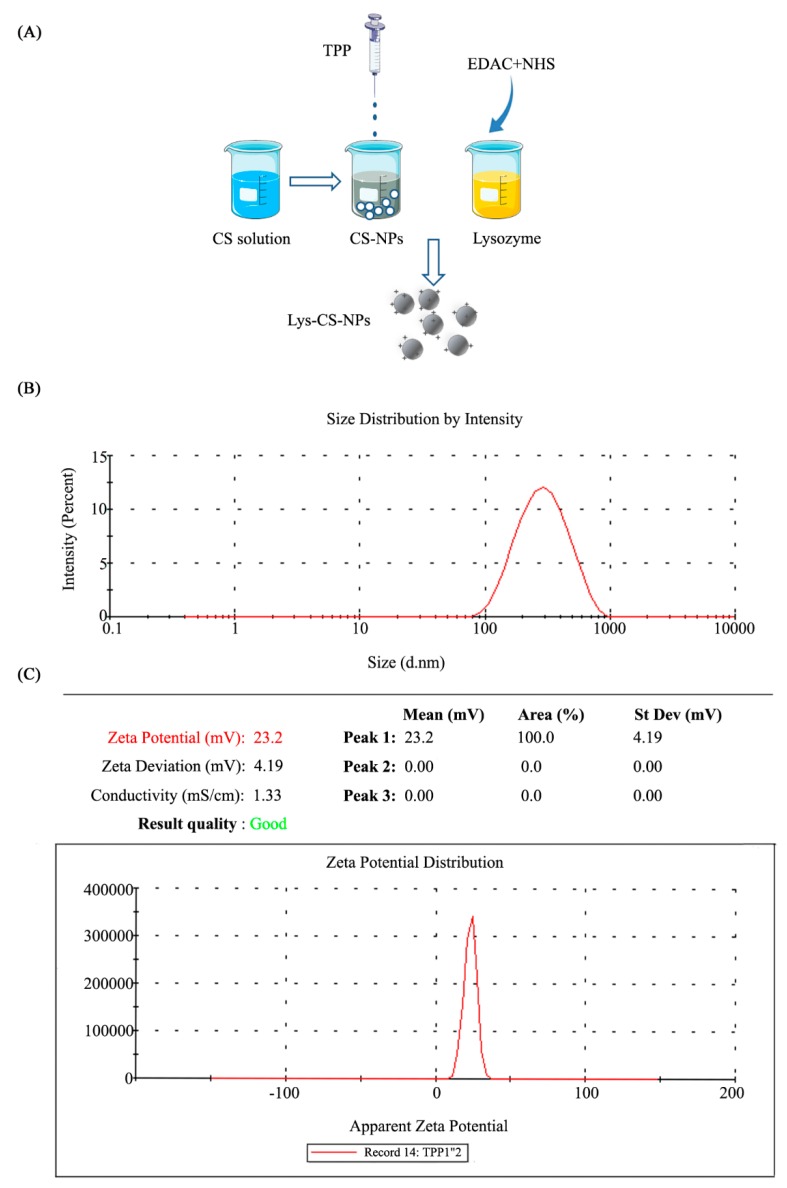
Characterization of nanoparticles: (**A**) schematic illustrations of the preparation process of Lys-CS-NPs; (**B**) particle size distribution of Lys-CS-NPs; and (**C**) zeta potential distribution of Lys-CS-NPs.

In this study, lysozyme was successfully immobilized in CS-NPs. Then, the morphology and size of synthetic Lys-CS-NPs was evaluated using TEM. CS-NPs exhibited a spherical shape and smooth surface, and the size of the Lys-CS-NPs was shown to be 100–300 nm (Figure S1). The size distribution further supported the results: the average diameter Lys-CS-NPs was 243.1 ± 2.1 nm, whereas the zeta potential was 22.8 ± 0.2 mV (Figure 1B,C).

**Figure 2:**
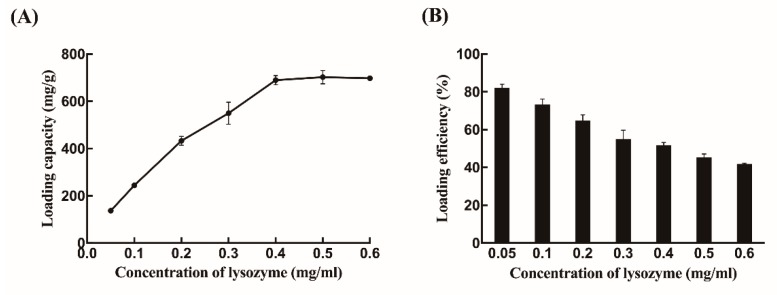
Analysis of lysozyme immobilized chitosan nanoparticles (Lys-CS-NPs): (**A**) lysozyme loading capacity of Lys-CS-NPs; and (**B**) lysozyme loading efficiency of Lys-CS-NPs.

Different concentrations of lysozyme were loaded into CS-NPs to identify the loading capacity (LC) and loading efficiency (%LE) of lysozyme. As shown in Figure 2A, the amount of encapsulated lysozyme apparently increased from 134.8 mg/g to 745.1 mg/g with 0.05–0.4 mg/mL concentration of lysozyme and then remained constant. Additionally, the %LE gradually decreased as the initial lysozyme loading increased. The %LE reached about 51.7% when the lysozyme concentration was 0.4 mg/mL (Figure 2B). CS-NPs were almost saturated by lysozyme at 0.4 mg/mL because the amounts of enzymes immobilized on the surfaces of CS-NPs was fixed. Thus, 0.4 mg/mL lysozyme, which showed a better encapsulated ability, was used as loading concentration for further study, as it was a more economical option.

### 2.2. Biochemical Characterizations of Free Lysozyme and Immobilized Lysozyme

**Figure 3:**
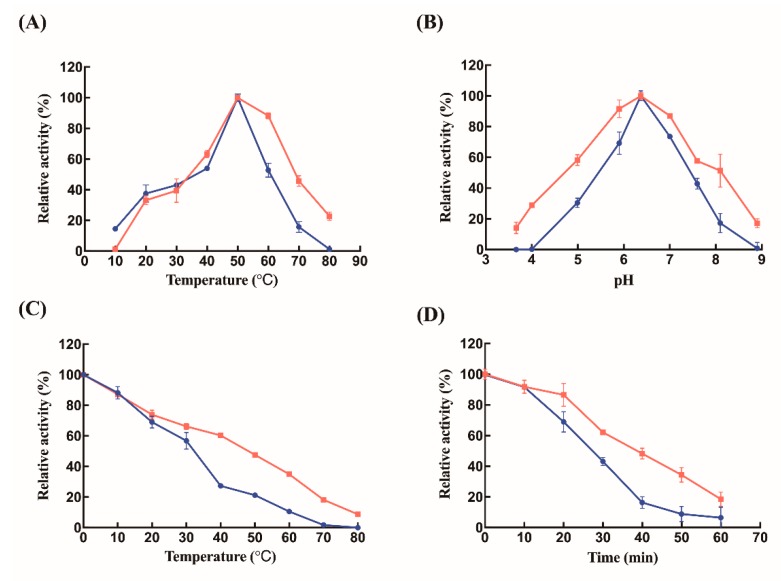
Biochemical characterization of free (blue circles) and immobilized (red squares) lysozyme. (**A**) Optimal temperature of the free lysozyme and immobilized lysozyme. The activities were determined in 20 mM sodium phosphate buffer (pH 6.5) for different temperatures from 10 to 80 °C. (**B**) The effect of pH on the activities of free lysozyme and immobilized lysozyme. Enzymatic activity assay was processed at 50 °C in various buffers as follows: 20 mM sodium acetate buffer (pH 3.7–5.0), 20 mM phosphate buffer (pH 6.0–7.0), and 20 mM glycine-NaOH buffer (pH 7.6–8.9). (**C**) Thermal stability of free lysozyme and immobilized lysozyme. Enzyme was incubated at various temperatures without substrate for 30 min and then its residual activities assayed at 50 °C and pH 6.5. (**D**) Thermo-stability of free and immobilized lysozyme at 37 °C for incubation at different times. The enzymatic activity of a fresh sample of free or immobilized lysozyme measured in 20 mM sodium phosphate buffer and at 50 °C was defined as 100%.

To determine the maximum enzymatic activity of the synthetic Lys-CS-NPs, free lysozyme with the same protein concentration was used as a control. The enzyme assay for Lys-CS-NPs demonstrated an effective activity around 70.8 ± 3.6% compared with free lysozyme. The immobilization process only slightly influenced the activity of lysozyme. In addition, the slightly decreasing activity of immobilized lysozyme may be correlated with the interaction between CS-NPs and proteins, which slightly changes the α-helix content of lysozyme and affects its structure and function. The biochemical characterizations of the free and immobilized lysozyme were studied herein. The free and immobilized lysozyme showed the optimum temperature at 50 °C (Figure 3A). Simultaneously, free lysozyme and immobilized lysozyme both exhibited maximum activities at pH 6.5 (Figure 3B). As a result, the process of immobilization on enzyme has little effect on the conditions, which can maximize its activities. However, the effect of immobilization on enzyme was reflected in its thermal stability. After treatment at different temperatures for 30 min, the enzymatic activities of free and immobilized lysozymes dropped with further increase of temperature, while the rate of reduction of activities of Lys-CS-NPs was lower than that of free enzyme (Figure 3C). Free lysozyme retained 27.3% and 21.3% of its initial activities following by incubation at 40 and 50 °C for 30 min, respectively, whereas Lys-CS-NPs maintained 60.3% and 47.6% relative activities under the same conditions, respectively. 

**Figure 4:**
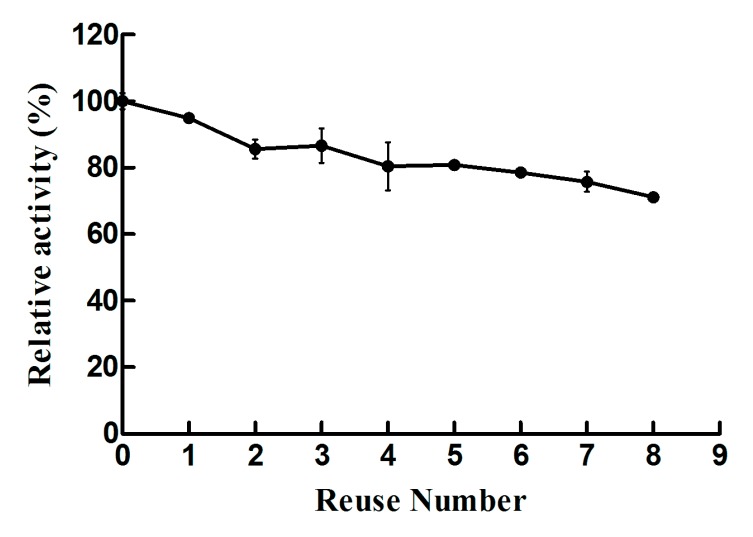
Reusability of Lys-CS-NPs. Activity for each cycle was compared with the initial activity.

The immobilized Lys-CS-NPs were washed with phosphate buffer (20 mM, pH 7.0) and resuspended into fresh substrate solution to start a new cycle following by testing its residual activities. As shown in Figure 4, immobilized lysozyme exhibited better reusability, whereas its activity decreased steadily with increasing number of using cycles and retained 71.1% of initial activity following the final eighth cycle. Free lysozyme was only use once and taken as a control. Evidently, immobilized Lys-CS-NPs exhibited superb reusability and excellent activity, due to the immobilized chitosan materials preventing the deactivation and leakage of enzyme.

### 2.3. Antibacterial Analysis

**Figure 5:**
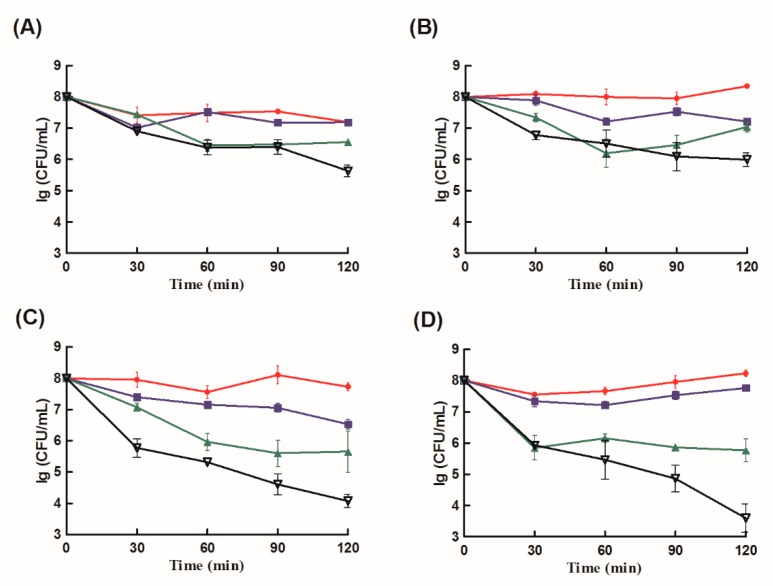
Antimicrobial activity kinetics of CS-NPs (red filled circles), free lysozyme (blue filled square), free lysozyme + CS-NPs complex (green filled triangle), and Lys-CS-NPs (black hollow triangle) for: *P. aeruginosa* (**A**); *K. pneumoniae* (**B**); *E. coli* (**C**); and *S. aureus* (**D**). lg, log10.

The bactericidal activities of CS-NPs, free lysozyme, a mixture of free lysozyme and CS-NPs, and Lys-CS-NPs against four strains (*P. aeruginosa*, *K. pneumoniae*, *E. coli*, and *S. aureus*) were determined (Figure 5). Lys-CS-NPs showed more pronounced effects toward *S. aureus* and *E. coli* at different times comparing with CS-NPs, free lysozyme, a mixture of free lysozyme and CS-NPs. For *P. aeruginosa* and *K. pneumoniae*, even if the changes of the four agents were not significantly different at the initial treatment, Lys-CS-NPs showed better bactericidal activities at 120 min. In the treatment of four strains, Lys-CS-NPs showed better effects than the mixture of free lysozyme and CS-NPs, indicating that it was the combination of both elements in the same system and not just the addition of the two effects that improve the efficacy.

The MIC values of different agents tested in vitro against different strains are shown in Table 1. The sensitivities of Gram-positive and Gram-negative bacteria to various reagents showed different values. Compared with the MIC values of CS-NPs and free lysozyme, Lys-CS-NPs and the mixture of lysozyme + CS-NPs exhibited superb antibacterial abilities, where the lower MIC can elicit the complete inactivation of strains. Lys-CS-NPs showed better effects than the simple mixture of free lysozyme + CS-NPs, indicating that integrating lysozyme into CS-NPs enhanced the sensitivities toward bacteria, especially toward Gram-positive bacteria (toward *S. aureus*: 10/64 mg/mL). 

The lysozyme loaded nanoparticles prepared by ionotropic gelation method with natural macromolecules chitosan evidenced excellent biocompatibility and sustained antibacterial activity compared with pure lysozyme according to the results of antibacterial assays. Therefore, in this research, it was postulated that it may be possible to improve antimicrobial activity against different bacteria by adsorption of lysozyme on nanocarriers prepared from natural biological macromolecules. 

## 3. Discussion

Lysozyme presents antimicrobial activity and is used in many fields, whereas chitosan exhibits antibacterial effect based on the interaction between chitosan and surface molecules. The combination of chitosan and lysozyme can theoretically exert better antibacterial ability. However, chitosan is known to be degraded by undergoing chain scission of its body by lysozyme via the hydrolysis of the β-1,4 N-acetylglucosamine units. However, the degree of deacetylation (DD) of chitosan plays important roles in degradation of chitosan with lysozyme, which leads to a complicated process. It has been proven that the original high DD of chitosan has fewer linkages between glycosamine and N-acetylglucosamine, which ultimately leads to less combination of hydrogen bonding with the lysozyme following by the changes of the lower flexibility of chitosan chains. In a previous study, the combination of NPs between the chitosan (DD: 92%) and lysozyme has successfully confirmed using glutaraldehyde as a crosslinker, whereas no signs of degradation of immobilized chitosan nanoparticles occurred in lysozyme solution during the incubation period [21]. Based on another study, free lysozyme was also integrated into CS-NPs based on the method of ionic gelation, demonstrating the structure of nanoparticles and the interactions between lysozyme and chitosan nanoparticles through various detection methods [22]. Thus, the outcomes of those studies indicate that the original CS with high DD was not degraded by lysozyme. In this study, chitosan with 93% DD was selected as the nanoparticle matrix. According to the results of TEM and DLS, Lys-CS-NPs was successfully assembled with appropriate, uniformly distributed particle size and stable zeta potential.

Moreover, enzymes are widely used in different fields due to their abilities of catalyzing difficult reactions, many of those reactions being currently impossible to be replaced by synthetic chemical reactions. The applications of the lysozymes are not without limitation, whereas the challenges are the inherent inabilities to continuously use, temperature instability, and the cost of replacing enzymes, which complicate the problem. The application of enzymatic engineering technology can improve enzymatic catalysis performance and thermo-stability, but the progress is quite difficult and requires specific screening methods and selection tools. Kawaguchi et al. prepared a lysozyme mutant with glycosylation at proper sites by yeast expression system, improving the thermodynamic stabilization of lysozyme but sacrificing the activity [23]. Upadhyay et al. applied a rapid identification of smart mutant libraries into protein stabilization, retaining catalytic activity, but the drawback is the absolute requirement of 3D protein structures [24]. Immobilization of enzymes on surfaces can possibly increase the stability of the enzyme and protect it from heat or chemical attack and prolong the time of use [25,26]. Immobilized enzyme in nanoparticles has proven to be a better arrangement as it improves the contact of substrate with bound enzymes by simultaneously reducing surface flow restrictions of the surface and shielding effect. It has been confirmed in some studies. Tarhan et al. prepared maltose-functionalized magnetic core–shell nanoparticles (Fe3O4@Au NPs) to effectively immobilized L-asparaginase (L-ASNase) and characterized their properties, being significantly improved acid–base tolerance and thermal stability and retention of 50% relative activity after reusing 13 cycles [27]. In addition, we also successfully immobilized a polysaccharide degrade enzyme, alginate lyase Aly08, into low molecule weight chitosan nanoparticles, which shows a great thermal stability and reusability compared to free Aly08 [28]. In this study, enzyme immobilization stabilized the enzyme conformation and helped to resist thermal denaturation and leaching, while the immobilized lysozyme showed better thermal stability and superb reusability. Lys-CS-NPs provided potential for future clinical applications. 

For patients in the hospital intensive care unit (ICU), the lung is the first infection site. Ventilator-associated pneumonia (VAP) caused by external intervention is the most common life-threatening hospital-acquired infection, while the pathogenic bacteria often include *K. pneumoniae* and *P. aeruginosa* [29]. The presence of the bacterial pathogen *S. aureus* promotes severe lesions including abscess formation and increases the risk of subsequent chronic respiratory infections with *P. aeruginosa* [30]. Additionally, recent studies have shown that *E. coli* also causes higher frequency of VAP [31]. Epaud et al. showed that delivery administration of lysozyme into lung through exogenous route can significantly enhance the effect of bacterial killing, even though endogenous lysozyme increases during acute pulmonary infection [32]. CS is a dispersibility enhancer of particles that can increase lung deposition, while CS-NPs have been shown to enhance mucosal adhesion or cellular absorption during pulmonary delivery [33,34]. In addition, the components in the Gram-negative bacteria biofilm (such as alginate and other acidic polysaccharides produced by *P. aeruginosa*) can be combined with the basic polysaccharide of chitosan, so that the CS-NPs can directly target the bacterial site, thereby increasing the targeted delivery of antibacterial agents. Lys-CS-NPs could be a potential candidate for subsequent pulmonary delivery for the treatment of bacterial infections.

For antibacterial lysozyme, it is essential to quickly and effectively exert antibacterial activity. For the antibacterial specificity of lysozyme, Gram-positive bacteria show more sensitivity than Gram-negative bacteria, whereas chitosan has antibacterial effect on both types of bacteria. Adding other bactericides or using other modification methods can broaden the antibacterial activity of lysozyme against different bacteria. Immobilization of lysozyme on chitosan is as one of those methods. Four different strains (one Gram-positive bacteria (*S. aureus*) and three Gram-negative bacteria (*P. aeruginosa*, *K. pneumoniae*, and *E. coli*)) were selected to evaluate the antibacterial capacity of the immobilized lysozyme. For free lysozyme, the hydrophobic outer membrane of Gram-negative bacteria often disrupts lysozyme activity. However, the mixture of free lysozyme and CS-NPs all showed lower MICs for the three Gram-negative bacteria, compared to free lysozyme or CS-NPs. The possible reason is that the electrostatic attraction between the cationic group in chitosan and the negatively charged components on the surface of the Gram-negative bacteria induces the permeability change of the shell, thereby enhancing the antibacterial effects of lysozyme. It is also essential to note that, for biofilm-forming strains, especially *P. aeruginosa*, the biofilm formation attributes to quorum sensing (QS), which mediates the secretion of exotoxin and exoenzymes (such as elastase, alginate, and extracellular polymeric substances), thus providing a protected environment for bacteria to resist various external damage [35]. However, Rubini et al. revealed QS signaling molecules inhibitory properties of chitosan, which inhibited biofilm formation of *P. aeruginosa* (PAO1) or few clinical isolated stains such as *P. aeruginosa* and *S. marcescens* [36]. This effect of chitosan is more conducive to the antibacterial activity of lysozyme. This is another reason the mixture of free lysozyme and chitosan nanoparticles has better antibacterial activity against *P. aeruginosa*. Immobilized lysozyme showed good antibacterial effect toward different bacteria, and the effects ware remarkably higher than the mixture of chitosan and free lysozyme or chitosan itself. The synergistic effects may be due to the increase in the average zeta potential of Lys-CS-NPs caused by the effects of interaction between lysozyme and CS-NPs, and the better surface charge density was responsible for the increasing interaction between Lys-CS-NPs and bacterial members, thus Lys-CS-NPs could cause more bacterial destruction. More studies are required to understand the antibacterial effect of Lys-CS-NPs in the lung environments.

## 4. Materials and Methods

### 4.1. Materials

Lysozyme were purchased from Solarbio (Beijing, China). *N*-(3-Dimethylaminopropyl)-*N*′-ethylcarbodiimide hydrochloride (EDAC), *N*-Hydroxysuccinimide (NHS) and Tri-polyphosphate (TPP) were purchased from Macklin (Shanghai, China). Chitosan (DD: 93%; viscosity: 20 mPa·s) were obtained from Aladdin (Shanghai, China). *P. aeruginosa*, *K. pneumoniae*, *S. aureus*, and *E. coli* were obtained from the Affiliated Hospital of Qingdao University (AHQU, Qingdao, China) and kept in frozen glycerol stock (maintained at −80 °C). Bacteria were cultured overnight in LB broth (Mdbio, Inc, Qingdao, China) at 37 °C for further assays. All other reagents used were analytical grade.

### 4.2. Preparation of Chitosan Nanoparticles

CS-NPs was prepared by ionotropic gelation method. Briefly, chitosan, 0.1 g, was dissolved in 100 mL acetic acid (1 mol/L) with continuous stirring and followed by adding 1 mol/L NaOH to get the final pH of the resulting mixture around 6.5. Then, the appropriate volumes of TPP (1 mg/mL) was slowly added dropwise into the prepared chitosan solution while stirring in room temperature (25 °C) for 1 h to obtain CS-NPs; the ratio of chitosan and TPP was 4:1. Further, the reaction mixture was then centrifuged twice at 10,000× *g* for 20 min with discarding the supernatant to collect the formed CS-NPs. The technique of carbodiimide chemistry was used for the formation of Lys-CS-NPs. Next, 10 mg/mL of lysozyme was mixed with 0.1 M EDAC and 0.1 M NHS to form the activated ester, and then the activated lysozyme was added into the CS-NPs at a final concentration of 0.05-0.50 mg/mL to obtain the Lys-CS-NPs. Finally, the reaction mixture was sonicated under ultrasonic conditions at 24 kHz, 8 W power, and 6 min with Ultrasonic Cell Disruption System (JY92-IIN, Ningbo, China) and ultrafiltrated with the Centricon centrifugal filter devices (molecular weight cut-off 50 kDa, Millipore, Darmstadt, Germany).

### 4.3. Evaluation of the Particle Properties

The shape and surface functionalization of immobilized nanoparticles Lys-CS-NPs were studied by TEM (JEM-2100, JEO, Tokyo, Japan). The samples were prepared by drying on the copper foil and then stained with phosphate tungsten acid, while the image was obtained under an accelerating voltage of 15.0 kV. The size, pdI, and zeta potential of the particle were characterized by DLS using Malvern platform (Malvern Nano ZSE, Malvern, UK). 

The loading capacity (LC), loading efficiency (%LE), and the remaining free lysozyme were determined spectrophotometrically by a Pearl-360 spectrophotometer (IMPLEN, Munich, Germany) with a wavelength of 280 nm. The concentration of lysozyme was analyzed with a calibration curve (Figure S2). LC and %LE were calculated as:

(1) $$\mathrm{LC}\;=\;\frac{\left(X-Y\right)}{Z}$$

(2) $$\mathrm{LE}\%\;=\;\frac{\left(X-Y\right)}{X}\times 100\%$$

X and Y represent the amount of lysozyme initially added to the formulation and the unload lysozyme, respectively, whereas Z represents the weight of the nanoparticles. All assays were performed in triplicate independently and the data are shown as mean ± standard deviation [37]. 

The release rate of lysozyme was determined by adding the Lys-CS-NPs in phosphate buffer solution (pH 7.4), whereas the Lys-CS-NPs was prepared by 0.5 mg/mL lysozyme. Lys-CS-NPs was mixed with 4 mL of 0.02 M phosphate buffer and the mixture was centrifuged and ultrasound 10 min to obtain the supernatant. The reaction system was replaced with equal fresh phosphate buffer solution followed by samples taken at the defined time points.

### 4.4. Enzyme Activity Assay

The activities of free lysozyme and Lys-CS-NPs were analyzed spectrophotometrically with some modifications [38,39]. Briefly, 0.3 mg/mL of *Micrococcus lysodeikticus* (900 µL) was mixed with the appropriately diluted enzyme solution (100 µL) at the optimum temperature (50 °C), and the reaction system was added to the quartz cuvette to further record the decrease of absorbance at 450 nm (A450). One unit (U) of enzyme activity was defined as the decrease of A450 by 0.001, under the above conditions. 

### 4.5. Characterization of Free and Immobilized Enzymes

Effects of temperatures on Lys-CS-NPs and free lysozyme activities were determined at 10–80 °C. The optimal pH for free lysozyme and Lys-CS-NPs was determined by assaying its activities at different pH using the following buffers: 20 mM Na_2_HPO_4_–citric acid buffer (pH 3.6–5.9), Na_2_HPO_4_–NaH_2_PO_4_ (pH 6.3–7.6), and glycine–NaOH (pH 8.1–8.9). The thermal stability was analyzed by measuring its residual activities after leaving it at various temperatures (10–80 °C) for 30 min. The residual activities were also measured after pre-incubating the free lysozyme and Lys-CH-NPs at 37 °C for 0–60 min to further characterize the thermal stability at specific temperature. The activities of lysozyme and immobilized lysozyme under different conditions were compared with those observed under their optimal reaction conditions, respectively, and then their relative activities were obtained.

### 4.6. Antibacterial Activity

Broth microdilution method was utilized to determine the MICs of antimicrobial agents (containing free lysozyme, CS-NPs, Lys-CS-NPs, and mixture of free lysozyme + CS-NPs). Bacterial cultures were obtained by pre-incubating *P. aeruginosa*, *K. pneumoniae*, *E. coli*, and *S. aureus* in LB broth at 37 ° C for 8 h and then diluting until an optical density reading of 0.1 was obtained, which meant bacterial density ≈1 × 10^8^ CFU/mL. Then, 100 µL LB broth and a certain concentration of antimicrobial agent solutions were added into a sterile 96-well plate, and the concentration of antibacterial agent solutions was serially diluted as 1:2. Equal volume of the four kind of bacteria culture (100 µL, 1 × 10^8^ CFU/mL) was seeded in the 96-well plates with mixing the four solutions and then incubated at 37 °C for 12 h. The control was set as bacteria culture (100 µL, 1 × 10^8^ CFU/ mL) adding into 100 µL LB broth. MICs of the agents were taken by the concentration at which no bacterial growth observed.

## 5. Conclusions

Lysozyme with key characteristics (e.g., isoelectric point and antibacterial activity) as a new and efficient antibacterial bioactive macromolecule has attracted increasing attention. However, its susceptibility to denaturation under physiological conditions has hindered its application as a therapeutic agent, thus loading it into polymer nanocarriers has become a promising strategy in clinical applications. In this study, lysozyme efficiently immobilized into chitosan nanoparticle showed a small particle size (243.1 ± 2.1 nm), positive zeta potential (22.8 ± 0.2 mV), and great loading efficiency when prepared by ionic gelation technique. Its physical–chemical and biological characterization was performed. No matter incubating at different temperatures or constantly at 37 °C for different times, the immobilized lysozyme all showed a higher residual activity than free lysozyme, which means a better thermal stability. Lys-CS-NPs also demonstrated a great reusability as they remained 71.1% activity after eight reuses compared with free lysozyme. The immobilized strategy enormously improved thermostability and reusability of lysozyme. Additionally, Lys-CS-NPs displayed excellent performance in inhibiting the growth of bacteria, especially *S. aureus* and *E. coli*. As for *P. aeruginosa* and *K. pneumoniae*, the immobilized lysozyme hah a better antibacterial ability at 120 min. In terms of MIC, Lys-CS-NPs all showed the minimum MIC value toward four strains. Therefore, according to the results of killing kinetics in vitro and MIC, lysozyme combined with CS-NPs owns more powerful bactericidal effect for both Gram-positive and -negative bacteria, whereas the effect is not the simple superposition effect stimulated by CS and lysozyme. Lys-CS-NPs could be a potential candidate for further development of lysozyme as an agent to control infections.

## Figures and Tables

**Table 1:** Inhibition of different strains as a planktonic population. MIC, minimum inhibitory concentration.

Concentration (mg/mL)	*P. aeruginosa*	*K. pneumoniae*	*E. coli*	*S. aureus*
CS-NPs	>10	10	10	10
Free lysozyme	>10	10	10/4	10/8
Free lysozyme + CS-NPs	10/2	10/2	10/16	10/32
Lys-CS-NPs	10/4	10/8	10/16	10/64

## Supplementary Materials

Supplementary Materials can be found at https://www.mdpi.com/1422-0067/21/5/1635/s1.

## Abbreviations

CS-NPs	chitosan nanoparticles
Lys-CS-NPs	lysozyme immobilized chitosan nanoparticles
TEM	transmission electron microscopy
DLS	dynamic light scattering
MIC	minimum inhibitory concentration
*P. aeruginosa*	*Pseudomonas aeruginosa*
*K. pneumoniae*	*Klebsiella pneumoniae*
*E. coli*	*Escherichia coli*
*S. aureus*	*Staphylococcus aureus*.
LC	loading capacity
%LE	loading efficiency
DD	degree of deacetylation
ICU	intensive care unit
VAP	ventilator-associated pneumonia
QS	quorum sensing
EDAC	*N*-(3-Dimethylaminopropyl)-*N*′-ethylcarbodiimide hydrochloride
NHS	*N*-Hydroxysuccinimide
TPP	Tri-polyphosphate
