# Supplementary material for: Enhancing the Thermo-Stability and Anti-Bacterium Activity of Lysozyme by Immobilization on Chitosan Nanoparticles
Source: Int J Mol Sci. 2020 Feb 27;21(5):1635. doi: 10.3390/ijms21051635 (PMC7084273; doi:10.3390/ijms21051635)
Supplement: Supplementary file 1 [file ijms-21-01635-s001.pdf]

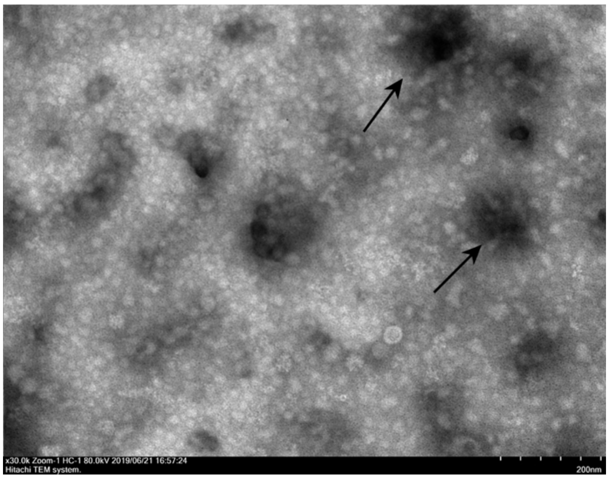

Figure S1. TEM image of Lys-CS-NPs. Arrows indicate nanoparticles

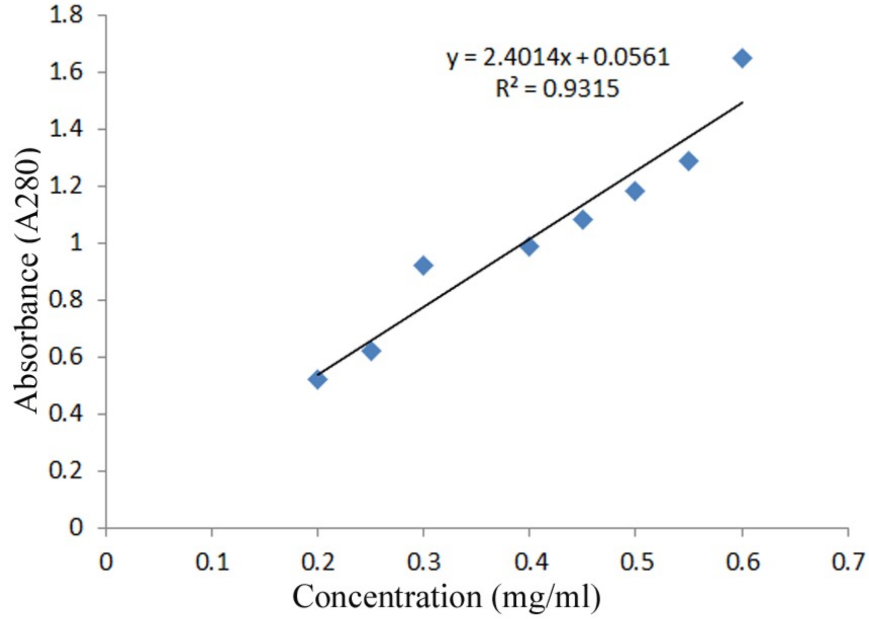

Figure S2. A calibration curve of the concentration of lysozyme.
